# Supplementary material for: OpenMM 7: Rapid development of high performance algorithms for molecular dynamics
Source: PLoS Comput Biol. 2017 Jul 26;13(7):e1005659. doi: 10.1371/journal.pcbi.1005659 (PMC5549999; doi:10.1371/journal.pcbi.1005659)
Supplement: S1 Source Code — (ZIP) [file pcbi.1005659.s002.zip › OpenMM-7.0.1-Source/docs/api-python/generated/simtk.openmm.app.forcefield.ForceField.html]

ForceField — OpenMM 7.0.1.dev-5e86c4f documentation


# ForceField¶

*class* `simtk.openmm.app.forcefield.``ForceField`(*\*files*)¶
:   A ForceField constructs OpenMM System objects based on a Topology.

    `__init__`(*\*files*)¶
    :   Load one or more XML files and create a ForceField object based on them.

        |  |  |
        | --- | --- |
        | Parameters: | **files** (*list*) – A list of XML files defining the force field. Each entry may be an absolute file path, a path relative to the current working directory, a path relative to this module’s data subdirectory (for built in force fields), or an open file-like object with a read() method from which the forcefield XML data can be loaded. |

    Methods

    |  |  |
    | --- | --- |
    | `__init__`(\*files) | Load one or more XML files and create a ForceField object based on them. |
    | `createSystem`(topology[, nonbondedMethod, ...]) | Construct an OpenMM System representing a Topology with this force field. |
    | `generateTemplatesForUnmatchedResidues`(topology) | Generate forcefield residue templates for residues in specified topology for which no forcefield templates are available. |
    | `getGenerators`() | Get the list of all registered generators. |
    | `getMatchingTemplates`(topology) | Return a list of forcefield residue templates matching residues in the specified topology. |
    | `getUnmatchedResidues`(topology) | Return a list of Residue objects from specified topology for which no forcefield templates are available. |
    | `loadFile`(file) | Load an XML file and add the definitions from it to this ForceField. |
    | `registerAtomType`(parameters) | Register a new atom type. |
    | `registerGenerator`(generator) | Register a new generator. |
    | `registerResidueTemplate`(template) | Register a new residue template. |
    | `registerScript`(script) | Register a new script to be executed after building the System. |
    | `registerTemplateGenerator`(generator) | Register a residue template generator that can be used to parameterize residues that do not match existing forcefield templates. |

    `loadFile`(*file*)¶
    :   Load an XML file and add the definitions from it to this ForceField.

        |  |  |
        | --- | --- |
        | Parameters: | **file** (*string or file*) – An XML file containing force field definitions. It may be either an absolute file path, a path relative to the current working directory, a path relative to this module’s data subdirectory (for built in force fields), or an open file-like object with a read() method from which the forcefield XML data can be loaded. |

    `getGenerators`()¶
    :   Get the list of all registered generators.

    `registerGenerator`(*generator*)¶
    :   Register a new generator.

    `registerAtomType`(*parameters*)¶
    :   Register a new atom type.

    `registerResidueTemplate`(*template*)¶
    :   Register a new residue template.

    `registerScript`(*script*)¶
    :   Register a new script to be executed after building the System.

    `registerTemplateGenerator`(*generator*)¶
    :   Register a residue template generator that can be used to parameterize residues that do not match existing forcefield templates.

        This functionality can be used to add handlers to parameterize small molecules or unnatural/modified residues.

        Caution

        This method is experimental, and its API is subject to change.

        |  |  |
        | --- | --- |
        | Parameters: | - **generator** (*function*) – A function that will be called when a residue is encountered that does not match an existing forcefield template. - **a residue without a template is encountered, the generator function is called with** (*When*) – |

        :param : success = generator(forcefield, residue)
        :type : :
        :param ``:
        :param where `forcefield is the calling ForceField object and residue is a simtk.openmm.app.topology.Residue object.:
        :param generator must conform to the following API:
        :param : Parameters

        > forcefield : simtk.openmm.app.ForceField
        > :   The ForceField object to which residue templates and/or parameters are to be added.
        >
        > residue : simtk.openmm.app.Topology.Residue
        > :   The residue topology for which a template is to be generated.
        >
        > success : bool
        > :   If the generator is able to successfully parameterize the residue, True is returned.
        >     If the generator cannot parameterize the residue, it should return False and not modify forcefield.
        >
        > The generator should either register a residue template directly with forcefield.registerResidueTemplate(template)
        > or it should call forcefield.loadFile(file) to load residue definitions from an ffxml file.
        >
        > It can also use the ForceField programmatic API to add additional atom types (via forcefield.registerAtomType(parameters))
        > or additional parameters.

        :type : :

    `getUnmatchedResidues`(*topology*)¶
    :   Return a list of Residue objects from specified topology for which no forcefield templates are available.

        Caution

        This method is experimental, and its API is subject to change.

        |  |  |
        | --- | --- |
        | Parameters: | **topology** (*Topology*) – The Topology whose residues are to be checked against the forcefield residue templates. |
        | Returns: | - **unmatched\_residues** (*list of Residue*) –   List of Residue objects from topology for which no forcefield residue templates are available.   Note that multiple instances of the same residue appearing at different points in the topology may be returned. - *This method may be of use in generating missing residue templates or diagnosing parameterization failures.* |

    `getMatchingTemplates`(*topology*)¶
    :   Return a list of forcefield residue templates matching residues in the specified topology.

        Caution

        This method is experimental, and its API is subject to change.

        |  |  |
        | --- | --- |
        | Parameters: | **topology** (*Topology*) – The Topology whose residues are to be checked against the forcefield residue templates. |
        | Returns: | - **templates** (*list of \_TemplateData*) –   List of forcefield residue templates corresponding to residues in the topology.   templates[index] is template corresponding to residue index in topology.residues() - *This method may be of use in debugging issues related to parameter assignment.* |

    `generateTemplatesForUnmatchedResidues`(*topology*)¶
    :   Generate forcefield residue templates for residues in specified topology for which no forcefield templates are available.

        Caution

        This method is experimental, and its API is subject to change.

        |  |  |
        | --- | --- |
        | Parameters: | **topology** (*Topology*) – The Topology whose residues are to be checked against the forcefield residue templates. |
        | Returns: | - **templates** (*list of \_TemplateData*) –   List of forcefield residue templates corresponding to residues in topology for which no forcefield templates are currently available.   Atom types will be set to None, but template name, atom names, elements, and connectivity will be taken from corresponding Residue objects. - **residues** (*list of Residue*) –   List of Residue objects that were used to generate the templates.   residues[index] is the Residue that was used to generate the template templates[index] |

    `createSystem`(*topology*, *nonbondedMethod=NoCutoff*, *nonbondedCutoff=Quantity(value=1.0*, *unit=nanometer)*, *constraints=None*, *rigidWater=True*, *removeCMMotion=True*, *hydrogenMass=None*, *\*\*args*)¶
    :   Construct an OpenMM System representing a Topology with this force field.

        |  |  |
        | --- | --- |
        | Parameters: | - **topology** (*Topology*) – The Topology for which to create a System - **nonbondedMethod** (*object=NoCutoff*) – The method to use for nonbonded interactions. Allowed values are   NoCutoff, CutoffNonPeriodic, CutoffPeriodic, Ewald, or PME. - **nonbondedCutoff** (*distance=1\*nanometer*) – The cutoff distance to use for nonbonded interactions - **constraints** (*object=None*) – Specifies which bonds and angles should be implemented with constraints.   Allowed values are None, HBonds, AllBonds, or HAngles. - **rigidWater** (*boolean=True*) – If true, water molecules will be fully rigid regardless of the value   passed for the constraints argument - **removeCMMotion** (*boolean=True*) – If true, a CMMotionRemover will be added to the System - **hydrogenMass** (*mass=None*) – The mass to use for hydrogen atoms bound to heavy atoms. Any mass   added to a hydrogen is subtracted from the heavy atom to keep   their total mass the same. - **args** – Arbitrary additional keyword arguments may also be specified.   This allows extra parameters to be specified that are specific to   particular force fields. |
        | Returns: | the newly created System |
        | Return type: | system |

    `__delattr__`¶
    :   x.\_\_delattr\_\_(‘name’) <==> del x.name

    `__format__`()¶
    :   default object formatter

    `__getattribute__`¶
    :   x.\_\_getattribute\_\_(‘name’) <==> x.name

    `__hash__`¶

    `__reduce__`()¶
    :   helper for pickle

    `__reduce_ex__`()¶
    :   helper for pickle

    `__repr__`¶

    `__setattr__`¶
    :   x.\_\_setattr\_\_(‘name’, value) <==> x.name = value

    `__sizeof__`() → int¶
    :   size of object in memory, in bytes

    `__str__`¶

High performance molecular simulation on GPUs


### Navigation

- Application Layer
  - Loaders and Setup
  - Representation and Manipulation
  - Simulation
  - Reporting Output
  - Extras
- Library Layer

©2015, Stanford University and the Authors.
|
Powered by Sphinx 1.3.1
& Alabaster 0.7.7
|
Page source
